# Inhibition of Histone Deacetylase 3 (HDAC3) Mediates Ischemic Preconditioning and Protects Cortical Neurons against Ischemia in Rats

**DOI:** 10.3389/fnmol.2016.00131

**Published:** 2016-11-28

**Authors:** Xiaoyu Yang, Qimei Wu, Lei Zhang, Linyin Feng

**Affiliations:** CAS Key Laboratory of Receptor Research, Shanghai Institute of Materia Medica, Chinese Academy of SciencesShanghai, China

**Keywords:** preconditioning (PC), HDAC3, MCAO, RGFP966, calpain

## Abstract

Brain ischemic preconditioning (PC) provides vital insights into the endogenous protection against stroke. Genomic and epigenetic responses to PC condition the brain into a state of ischemic tolerance. Notably, PC induces the elevation of histone acetylation, consistent with evidence that histone deacetylase (HDAC) inhibitors protect the brain from ischemic injury. However, less is known about the specific roles of HDACs in this process. HDAC3 has been implicated in several neurodegenerative conditions. Deletion of HDAC3 confers protection against neurotoxicity and neuronal injury. Here, we hypothesized that inhibition of HDAC3 may contribute to the neuronal survival elicited by PC. To address this notion, PC and transient middle cerebral artery occlusion (MCAO) were conducted in Sprague-Dawley rats. Additionally, primary cultured cortical neurons were used to identify the modulators and effectors of HDAC3 involved in PC. We found that nuclear localization of HDAC3 was significantly reduced following PC *in vivo* and *in vitro*. Treatment with the HDAC3-specific inhibitor, RGFP966, mimicked the neuroprotective effects of PC 24 h and 7 days after MCAO, causing a reduced infarct volume and less Fluoro-Jade C staining. Improved functional outcomes were observed in the neurological score and rotarod test. We further showed that attenuated recruitment of HDAC3 to promoter regions following PC potentiates transcriptional initiation of genes including *Hspa1a*, *Bcl2l1*, and *Prdx2*, which may underlie the mechanism of protection. In addition, PC-activated calpains were implicated in the cleavage of HDAC3. Pretreatment with calpeptin blockaded the attenuated nuclear distribution of HDAC3 and the protective effect of PC *in vivo*. Collectively, these results demonstrate that the inhibition of HDAC3 preconditions the brain against ischemic insults, indicating a new approach to evoke endogenous protection against stroke.

## Introduction

Brain ischemia, induced by transient or permanent interruption of the blood supply, is a major cause of mortality and morbidity worldwide (Johnston et al., 2009; Feigin et al., 2014). The mechanisms of neuronal death in stroke are complex; involving cell membrane depolarization, free radical generation, excitotoxicity, and neuroinflammation (Dirnagl et al., 1999; Moskowitz et al., 2010). Despite extensive efforts, therapeutic strategies focusing on the pathological signaling cascades have not been successful (Moretti et al., 2015). Effective treatments for ischemic brain damage remain one of the major unfulfilled medical needs of clinical care (Fisher and Saver, 2015). Ischemic PC is an approach in which a sub-lethal ischemic exposure evokes endogenous protection against a subsequent, more severe ischemic insult. Not only can this therapy be applied in individuals, induced ischemic tolerance is also a strategy to obtain insights into neuroprotective mechanisms (Dirnagl et al., 2009; Stevens et al., 2014). PC stimuli may be identified by diverse sensors and transducers, and consequently initiate protection such as temporal gene profiles resistant to typically lethal ischemic insults by effectors.

Different genomic profiles and epigenetic reprogramming of the brain have been reported between ischemic PC and ischemic injury (Stenzel-Poore et al., 2003; Thompson et al., 2013). Thereby identification of epigenetic determinants of PC is the key for pharmacological manipulation to evoke conditioning. *In vivo* studies have shown that pan-HDAC inhibitors protected the brain from ischemic injury, by elevating the severe decrease of histone acetylation (Chuang et al., 2009; Langley et al., 2009). The treatment of myocardial ischemia with HDAC inhibitors triggers the PC effects against ischemia/reperfusion injury. Likewise, studies in retina and brain ischemia revealed an elevation of histone acetylation following PC, which may be associated with regulation of the deacetylase activity of HDAC or HAT recruitment (Yildirim et al., 2014; Fan et al., 2016). These studies have raised the hypotheses that HDACs might converge in the conditioning signaling pathways. However, considering the significant effects of HDAC inhibitors against ischemia, less is known about the specific role of HDACs in brain PC.

HDAC3, a homologue of Rpd3 from budding yeast, has been linked to neurotoxicity in several neuropathological conditions (Butler and Bates, 2006; Yang and Seto, 2008). In *Caenorhabditis elegans* knock-down of the homolog of HDAC3, HDA-3, suppressed Htn-Q150 toxicity in a model of Huntington’s disease (Bates et al., 2006). In rat cerebellar granule neurons, mutant *Htt* disrupted the sequestration of HDAC3 and the liberation of HDAC3 resulted in neurotoxic activity (Bardai et al., 2013). Additionally, suppression by HDAC3 shRNA protected cerebellar granule neurons against a low-potassium insult, while overexpression of HDAC3 promoted the death of neurons (Bardai and D’Mello, 2011). Conditional knock-out of *Hdac3* in retinal ganglion cells displayed a significant amelioration of nuclear atrophy and reduction in cell death induced by optic nerve injury (Schmitt et al., 2014).

Given the properties of HDAC3 in neurodegeneration, we speculate whether inhibition of HDAC3 contributes to the neuronal survival elicited by PC. We first investigated histone acetylation and class I HDAC subcellular localization following PC. We found that PC-induced acetyl-histone 3 Lysine 9 (H3K9ac) elevation was accompanied by reduced HDAC3 nuclear localization in cortical neurons. Based on this, efficient and specific pharmacological inhibition of HDAC3 *in vivo* and knock-down of HDAC3 *in vitro* in models of ischemia were performed. The results showed that specific inhibition of HDAC3 could precondition the brain against ischemic injury 24 h and 7 days after MCAO *in vivo*, via the initiation of a gene-expressing program associated with neuroprotection. Furthermore, we identified that calpains were implicated in the cleavage of HDAC3, which blocked the nuclear distribution of HDAC3. Overall, the results demonstrate the importance of HDAC3 in the process of PC, providing a new pharmacological approach to evoke protection due to ischemic conditioning.

## Materials and Methods

### Animals

Adult male Sprague-Dawley rats weighing 250–280 g were used in this study (Laboratory Animal Center, Chinese Academy of Sciences, Shanghai, China). All experimental procedures were performed in accordance with the Guide for the Care and Use of Laboratory Animals (National Institutes of Health) and approved by the Institutional Animal Care and Use Committee of Shanghai Institute of *Materia Medica*.

### MCAO and Ischemic Preconditioning

All animals were acclimatized for 2 weeks before pharmacological treatment or surgery. The induction of transient focal cerebral ischemia was performed as previously described (Longa et al., 1989), with minor modifications. Briefly, the animals were anesthetized with chloral hydrate (400 mg/kg, i.p.). A 4-0 monofilament nylon surgical suture (Sunbio Biotech Co. Ltd., Beijing, China) with a rounded tip was introduced through the external carotid artery to the internal carotid artery and advanced up to 18–20 mm to block the middle cerebral artery. After a period of occlusion, 5 min for ischemic PC and 90 min for injurious ischemia, the suture was withdrawn to allow reperfusion. Regional cerebral blood flow was monitored throughout surgery using a laser Doppler (moorVMS-LDF2, Axminster, UK) to exclude rats that showed a cerebral blood flow (CBF) reduction of less than 80%. Rectal temperature was monitored and the core body temperature was maintained at 37 ± 0.5°C by a feed-back-controlled heat pad (Hugo Sachs Elektronik, March-Hugstetten, Germany). Rats were transferred back to their cages after recovery from anesthesia and rested in home cages before sacrifice. Sham operation were conducted using an identical procedure but without the insertion of a filament.

### Experimental Design

Rats were randomly grouped (*n* = 8–14 per group) and subjected to different MCAO and pharmaceutical treatments as follows (Supplementary Figures S2A,B):

Sham group: Sham surgery was performed on day 1 and at the same time on day 2. Rats received vehicle injections at the same time as the RGFP966 group or the calpeptin group below.
MCAO group: Rats underwent sham operation on day 1 and 90 min of MCAO on day 2. Rats received vehicle injections at the same time as RGFP966 group or calpeptin group below.
PC group: Rats were subjected to 5 min of MCAO to induce ischemic PC on day 1, and 90 min of MCAO on day 2.
RGFP966 group: Rats were treated with RGFP966 (7.5 mg/kg, i.p) when subjected to a sham operation. The second RGFP966 was injected 6 h prior to the MCAO of 90 min on day 2.
Calpeptin group: Rats were treated with calpeptin (125 μg/kg, i.p) 6 h before surgery, and then underwent the same procedure as the PC group.
Rats were sacrificed 24 h or 7 days after injurious MCAO.

### Pharmacological Treatment

Drugs were administrated at a volume of 5 ml/kg. RGFP966 (S7229, Selleckchem, Houston, TX, USA) was dissolved in DMSO, and diluted in a vehicle of 100 mM sodium acetate (PH 5.4) and 30% (wt/vol) hydroxypropyl-beta-cyclodextrin, with the final DMSO less than 10% (vol/vol). Calpeptin (C8999, Sigma–Aldrich, St Louis, MO, USA) was dissolved in DMSO and diluted in saline. Vehicles were obtained by identical procedures without drugs. The doses were determined based on pervious report to obtain proper concentrations in the brain (Malvaez et al., 2013; Samantaray et al., 2015).

### Infarct Volume Quantification

The cerebral infract volumes were measured using TTC (T8877, Sigma–Aldrich) staining. Rat brains were harvested and sectioned into 2 mm thick coronal sections 24 h or 7 days after MCAO. Then sections were stained with 1% TTC at 37 °C for 5 min, and fixed in 4% paraformaldehyde (PFA) solution for 48 h. The images were captured and analyzed using Image-Pro Plus 7.0 (Media Cybernetics, Silver Spring, MD, USA). The infarct volume (%) for the brain was calculated with the following formula: (the volume of the contralateral hemisphere – the volume of the non-lesioned ipsilateral hemisphere)/(the volume of the contralateral hemisphere × 2). The ipsilateral hemisphere underwent occlusive treatment in the MCA, while the contralateral hemisphere did not. Investigators were kept blind to the treatment assignments.

### Neurological Function Analysis

The neurological deficits score after surgery was evaluated according to validated scoring analyses with minor modifications (Longa et al., 1989): 0, no observable deficit; 1, unable to fully stretch the forelimb of the right side (the left MCA was occluded in this study); 2, decreased resistance to lateral push, circling to the right side but normal gesture at rest; 3, unilateral rolling to the right side; 4, occasionally leaning to the right side, declined locomotor activity; 5, unable to sustain normal gesture at rest, unresponsive. Investigators were kept blind to treatment assignments.

### Rotarod Test

Rats were trained and tested on rotarod (IITC Life Science, Woodland Hills, CA, USA) which gradually accelerated from 4 to 40 rpm over 5 min (Hunter et al., 2000). The latency to fall was recorded as the time before rats fell off the rod or gripped and spun around for two successive revolutions. Rats underwent three training trails a day for 3 days. The base line control was recorded 1 day before the surgery. The mean latency was obtained from 3 trials with 30 min interval on the testing day. Investigators were kept blind to treatment assignments.

### Primary Culture of Rat Cortical Neurons and Treatment

Primary cortical neurons were prepared from embryonic E17 Sprague-Dawley rats and cultured with Neurobasal medium and B27 supplement (Thermo Fisher Scientific, USA) as described previously (Xie et al., 2009). Neurons were cultured at 37°C in a humidified 5% CO_2_ atmosphere and the medium was replaced by fresh every 3 days. Neurons at 7 DIV were used for experimentation. In the OGD treatment, the glucose-free Dulbecco’s Modified Eagle Medium (DMEM) (Thermo Fisher Scientific) was bubbled with 95% N_2_/5% CO_2_. The chamber was pre-warmed, humidified, and flushed with 95% N_2_/5% CO_2_ at 3 liter/min for 5 min. The oxygen content was under 0.50% (v/v) throughout the experimental period detected with oxygen monitor (Jiande Analytical Instrument, Hangzhou, China). For PC treatment, the culture medium was replaced with and cells were placed glucose-free DMEM in an experimental hypoxia chamber for 45 min for neurons or 1.5 h for PC12. The cells were then removed from the chamber and cultured under normal conditions for the indicated periods (see figure legends). For the OGD/R model, neurons were cultured in glucose-free DMEM in a hypoxia chamber for 90 min and returned to normal culture conditions for 24 h. The control cells were cultured in a medium with glucose in a normal oxygen-condition incubator for the same time periods.

### Tissue Preparation

After anesthetization, brains were immediately removed. Brain tissue were obtained by coronal sectioning and harvested along the core region of occlusion underlying the middle cerebral artery. The tissues were immediately frozen in liquid nitrogen, and stored at -80°C.

### Western Blot

The brain tissue or cultured cells were lysed in a RIPA buffer with proteinase and phosphatase inhibitor cocktail (Sigma–Aldrich) for whole cell protein. The nuclear and cytoplasmic fractions were extracted with NE-PER Nuclear and Cytoplasmic Extraction Reagents Kit (Pierce, Rockford, IL, USA) following the manufacturer’s instruction. The protein concentration was quantified using a BCA assay kit (Thermo Fisher Scientific). Samples were separated by sodium dodecyl sulfate-polyacrylamide gel electrophoresis (SDS-PAGE) and transferred to PVDF membranes (Merck-Millipore, Bedford, MA, USA). Blots were then probed with specific antibodies: anti-H3K9ac (ab12179, Abcam, Cambridge, MA, USA), anti-HDAC1 (WH0003065M2, Sigma–Aldrich), anti-HDAC2 (ab16032, Abcam), anti-HDAC3 (ab47237, Abcam), anti-lamin A/C (2032, Cell Signaling Technology, Beverly, MA, USA), anti-β-actin (A8481, Sigma–Aldrich), anti-Bcl-xL (2764, Cell Signaling Technology), anti-Prdx2 (10545-2-AP, Proteintech Group, Chicago, IL, USA), anti-HSP70 (sc-32239, Santa Cruz, Dallas, TX). After incubation with HRP-conjugated secondary antibodies (Santa Cruz), chemiluminescence signals were detected with ECL reagents (GE Health, Little Chalfont, UK).

### Histological and Immunohistochemical Assessment

After anesthesia with chloral hydrate, rats were injected transcardially with ice-cold 4% PFA in PBS. Brains were post-fixed in PFA for 24 h and dehydrated with 30% (wt/vol) sucrose for 48 h. Next, 25 μm coronal slices were obtained with a Leica freezing microtome. Primary antibodies used in immunofluorescence staining were: anti-H3K9ac (ab12179, Abcam), anti-HDAC3 (ab47237, Abcam) and anti-MAP2 (ab11267, Abcam). The sections were incubated with 300 nM 4,6-diamidino-2-phenylindole (DAPI, Sigma), Alexa Fluor 488 and Alexa Fluor 555 secondary antibodies (Thermo Fisher Scientific) for 45 min at 25°C. An Olympus FV1000 confocal laser scanning microscope was applied for acquisition of fluorescence images. 3,3′-diaminobenzidine immunostaining was performed with primary antibodies against: HSP70 (sc-32239, Santa Cruz), following with SignalStain Boost IHC Detection Reagent and visualized with SignalStain DAB Substrate Kit (Cell Signaling Technology). Images were acquired using a Leica microscope. Fluoro-Jade C (FJC) staining was performed according to the protocol provided by the manufacturer (Chemicon, Merck-Millipore). Images were obtained using an Olympus FV1000 confocal microscope. For histological quantification of H3K9ac intensity and FJC positive cells, images were obtained from two random 20× fields of the cortical area in three coronal sections (Interaural 10.60 mm/Bregma 1.60 mm, Interaural 9.20 mm/Bregma 0.20 mm, Interaural 7.12 mm/Bregma -1.88 mm) (Supplementary Figure S2C). Average intensities or cell counts were calculated from 5 rats per group with Image Pro Plus 7.0. Investigators were kept blind to treatment assignments.

### Co-immunoprecipitation (Co-IP) Assay

Cell lysates were generated by incubating cells with an NP-40 lysis buffer with proteinase inhibitor cocktail on ice and then centrifuged at 10000 *g* for 10 min. Immunoprecipitation was carried out by incubating the lysate with the corresponding antibodies: anti-HDAC3 (ab47237, Abcam), anti-calpain1 (ab28258, Abcam), anti-calpain2 (ab39165, Abcam) and anti-HA (3724, Cell Signaling Technology) with slow agitation overnight at 4°C. Next, 20 μl PureProteome Protein A/G Mix Magnetic Beads (Merck-Millipore) was added and incubated with slow agitation for 4 h at 4°C. Immune complexes were washed extensively, boiled in SDS-sample buffer and analyzed using SDS-PAGE.

### Quantitative Reverse Transcription Polymerase Chain Reaction (qRT-PCR)

Total RNA was extracted from cortical neurons after treatment using TRIzol reagent (Thermo Fisher Scientific). Next, 1 μg total RNA was converted to cDNA using a reverse transcription kit (Takara, Cat^#^ RR047A). After the RT reaction, cDNA was used for subsequent real time PCR (SYBR Premix Ex TaqII Kit) following the manufacturer’s protocol. The qPCR and data collection were carried out on ABI 7500 real-time PCR system (Applied Biosystems, Foster City, CA, USA). The results relative to the quantitation value for each target gene were expressed as 2^-ΔΔCt^. All PCR primers are shown in Supplementary Table S1.

### Chromatin Immunoprecipetition and Real-Time PCR

Chromatin immunoprecipetition was carried out according to the protocol of the EZ-Magna ChIP kit (17-10086, Millipore). Briefly, the nuclear content of cortical neurons was extracted and the chromatin inside was sonicated into fragments. Fragmented chromatin was incubated and immunoprecipitated using anti-H3K9ac antibody (17-609, Millipore) and anti-HDAC3 antibody (17-10238, Millipore). After the de-crosslink, immunoprecipitated DNA and whole-cell extract DNA were then eluted and subjected to real-time PCR analysis using SYBR Premix Ex TaqII Kit (Takara, Shiga, Japan). All PCR primers are shown in Supplementary Table S2.

### Plasmid Constructs and Transfection

Full-length human HDAC3 cDNA was synthesized by GENEWIZ (South Plainfield, NJ, USA) and ligated into the expression vector, HA-pcDNA3.0. C-terminus-truncated construct HDAC3 mutant (ΔC-HDAC3, 1-312) was amplified with primers: 5′GATATCGATGGCCAAGACCGTGGCGTATTTC3′ and 5′CTCGAGTTAAGATGTTTCATATGTCCAGCACCG3′ using full length HDAC3 cDNA as the template. Transient transfection was conducted using the FuGENE HD transfection reagent (Promega, Madison, WI, USA) according to the manufacturer’s instructions. siRNA-HDAC3 (s136736, Dharmacon) was transfected into cortical neurons by the FuGENE HD transfection reagent. The silencing effect of protein expression was confirmed by western blot analysis.

### Cell Viability

Neuron viability was assessed using the MTT assay as described previously. Briefly, MTT solution (0.5 mg/ml) was added to each well and incubated with cells for an additional 3 h at 37°C. The formazan was then dissolved with DMSO and the absorbance at 595 nm was read by a microplate reader (NOVOstar, BMG LABTECH, Offenburg, Germany).

### Statistical Analysis

Quantifications and statistical analysis were carried out using GraphPad Prism 6 (Graphpad Software, San Diego, CA, USA). All values are presented as means ± SEM. Differences between groups were compared with a one-way ANOVA followed by group comparisons using a *post hoc* Bonferroni test or two-tailed Student’s *t*-test. Neurological deficit scores were analyzed using the Kruskal–Wallis non-parametric test followed by Dunn’s multiple comparisons test.

## Results

### Acetylation of H3K9 Increased in Cortical Neurons in Preconditioning, Accompanied by Reduction of Nuclear Localization of HDAC3

**FIGURE 1:**
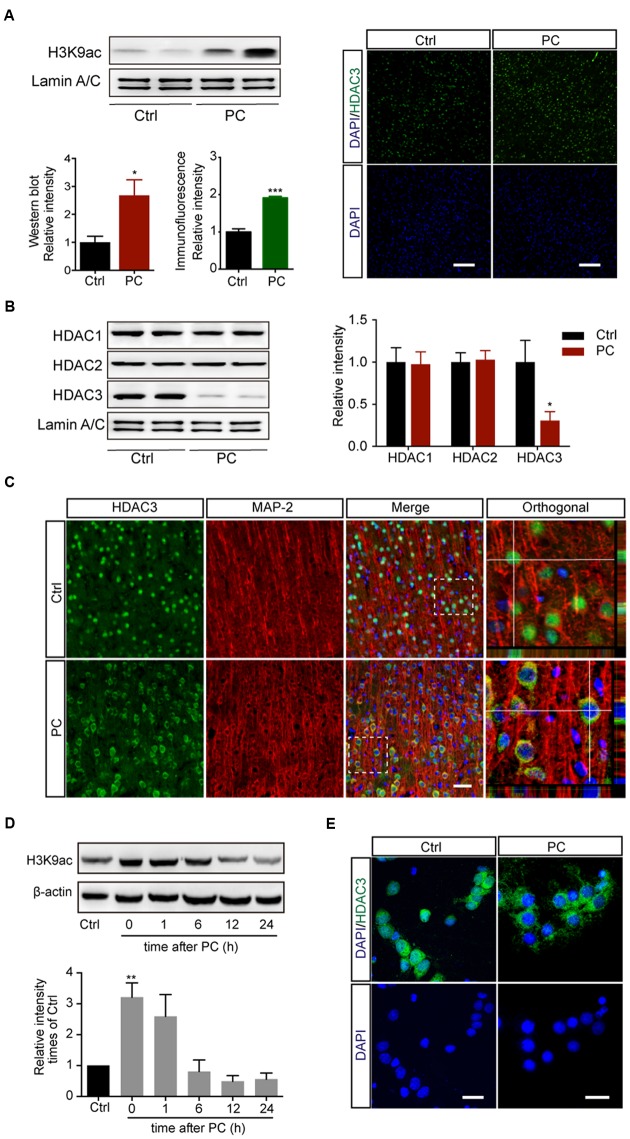
**Preconditioning induced the elevation of acetyl-H3K9 levels and reduced HDAC3 nuclear localization in rat cortical neurons *in vivo* and *in vitro*.** Rats underwent 5 min PC treatment followed by 2 h reperfusion **(A–C)**. **(A)** Acetyl-H3K9 levels in the cortex by immunoblotting (Left, *n* = 5) and immunohistochemistry (Right, *n* = 5). ^∗^*p* < 0.05, ^∗∗∗^*p* < 0.001 Student’s *t*-test. Scale bar: 100 μm. **(B)** Quantification of class I HDAC subtypes in nuclear lysates (*n* = 4–6). ^∗^*p* < 0.05, Student’s *t*-test. **(C)** Representative images of HDAC3 subcellular localization following sham or PC treatment *in vivo*. Scale bar: 50 μm. **(D)** Primary cultured cortical neurons underwent PC for 45 min followed by reoxygenation and restored energy supply for indicated period. Levels of H3K9 acetylation were assessed with whole cell lysates. Data are mean ± SEM. ^∗∗^*p* < 0.01 versus control, ANOVA. **(E)** Representative images of HDAC3 subcellular localization in rat cortical neurons after PC. Scale bar: 25 μm.

Histone acetylation was shown to be differentially regulated depending on the duration of ischemia. While injurious ischemia led to a severe decline of histone acetylation in stroke models (Supplementary Figure S1A) (Ren et al., 2004; Faraco et al., 2006; Kim et al., 2007), PC for 5 min followed by 2 h reperfusion in rats induced increased levels of acetyl-H3K9 in the cortex, as assessed with both immunoblotting (*p* < 0.05, *n* = 5) and immunohistochemistry (*p* < 0.001, *n* = 5) (**Figure 1A**). We next measured levels of expression of the three class I HDAC subtypes with which the deacetylation of histone lysine residuals is largely associated. Western blot assay on nuclear lysates revealed that the levels of HDAC3, but not HDAC1 or HDAC2, were reduced in the nuclei following PC (*p* < 0.05, *n* = 4–6) (**Figure 1B**). To further determine the involvement of HDAC3 in PC, the subcellular localization of HDAC3 was assessed with immunofluorescence. The results showed that HDAC3 was distributed both in the nucleus and cytoplasm in cortical neurons before PC, while it was localized mainly in the cytoplasm following PC treatment (**Figure 1C**). Meanwhile, the modulation of histone acetylation (**Figure 1D**) and the nuclear reduction of HDAC3 following PC were verified with primary cortical neuron cultures (**Figure 1E**, Supplementary Figures S1C,D). These results indicated that a diminished recruitment of HDAC3 to the genomic arena might contribute to the modulation of the histone acetylation profile following PC.

### Inhibition of HDAC3 Mimicked the Neuroprotective Effects of PC *In vivo*

**FIGURE 2:**
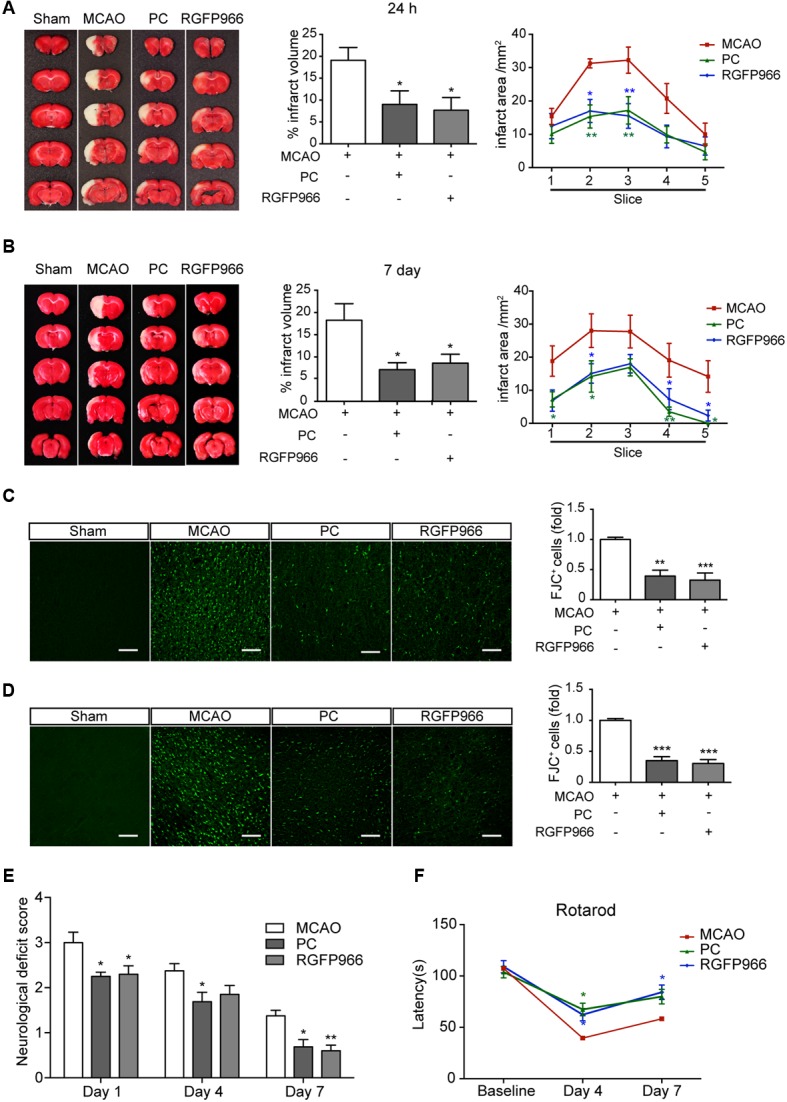
**RGFP966 protected the brain from ischemic injury *in vivo*.** Infarct measurements were performed 24 h (*n* = 7–9) **(A)** and 7 days (*n* = 8–10) **(B)** after MCAO. Representative images of TTC staining (Left), infarct volume percentage (Middle) and infarct area for each slice (Right) from each group. ^∗∗^*p* < 0.01, ^∗^*p* < 0.05 versus MCAO group, ANOVA. Representative images of Fluoro-Jade C staining in each group. The histogram shows the numbers of degenerating neurons in the cortex 24 h **(C)** or 7 days **(D)** after MCAO (over 30 slices from five rats per group). ^∗∗^*p* < 0.01, ^∗∗∗^*p* < 0.001 versus MCAO group, ANOVA. Functional outcomes were assessed using the neurological deficits score (day 1, 4, and 7 after MCAO) **(E)** and rotarod test (day 4 and 7 after MCAO) **(F)** (*n* = 8–10). ^∗^*p* < 0.05, ^∗∗^*p* < 0.01 versus MCAO group, ANOVA.

Given the changes in redistribution of HDAC3 following PC, we wondered whether inhibition of HDAC3 mimics the neuroprotective effect of PC. Rats with different treatments were subjected to MCAO (Supplementary Figure S2A). RGFP966, a known HDAC3-specific inhibitor, was injected 24 and 6 h prior to MCAO. The RGFP966-treated group exhibited significantly reduced infarct volume compared with the vehicle-treated group 24 h (7.68 ± 2.90% versus 19.09 ± 2.91%, *p* < 0.05, *n* = 7–9) and 7 days (8.62 ± 2.00% versus 18.25 ± 3.75%, *p* < 0.05, *n* = 8–10) after reperfusion. Furthermore, the anterior-posterior analysis of infarct distribution revealed that the infarction mostly occupied the striatum and temporal cortex, which was decreased in slices 2, 3, 4 in the PC and RGFP966 group (**Figures 2A,B**). Brain coronal sections obtained 24 h and 7 days after MCAO were stained with FJC to label degenerating neurons (**Figures 2C,D**). Higher levels of FJC staining were observed in the MCAO group compared to with the Sham group, and significantly decreased labeling with FJC in the RGFP966-treated group compared with the MCAO group in both 24 h and 7 days assessments (*n* = 5). Furthermore, we analyzed the counterstain of FJC with GFAP or Iba with brain sections obtained 24 h after MCAO. The schematic brain section and outlined box showed the area where images were acquired in the ipsilateral hemisphere (Supplementary Figure S5A). Activated astrocytes and microglia were observed in MCAO group, while less activation in PC and RGFP966 group (Supplementary Figures S5B,C). Meanwhile, neurological function was evaluated using the neurological deficit score and rotarod test. Rats treated with RGFP966 displayed lower neurological deficit scores (*n* = 8–10) and improved rotarod performance (*n* = 8–10) throughout 7-day test compared with the Sham group (**Figures 2E,F**).

### Attenuated Recruitment of HDAC3 to Promoter Regions Following PC Potentiated Transcriptional Initiation of Oxidation Relative Genes

**FIGURE 3:**
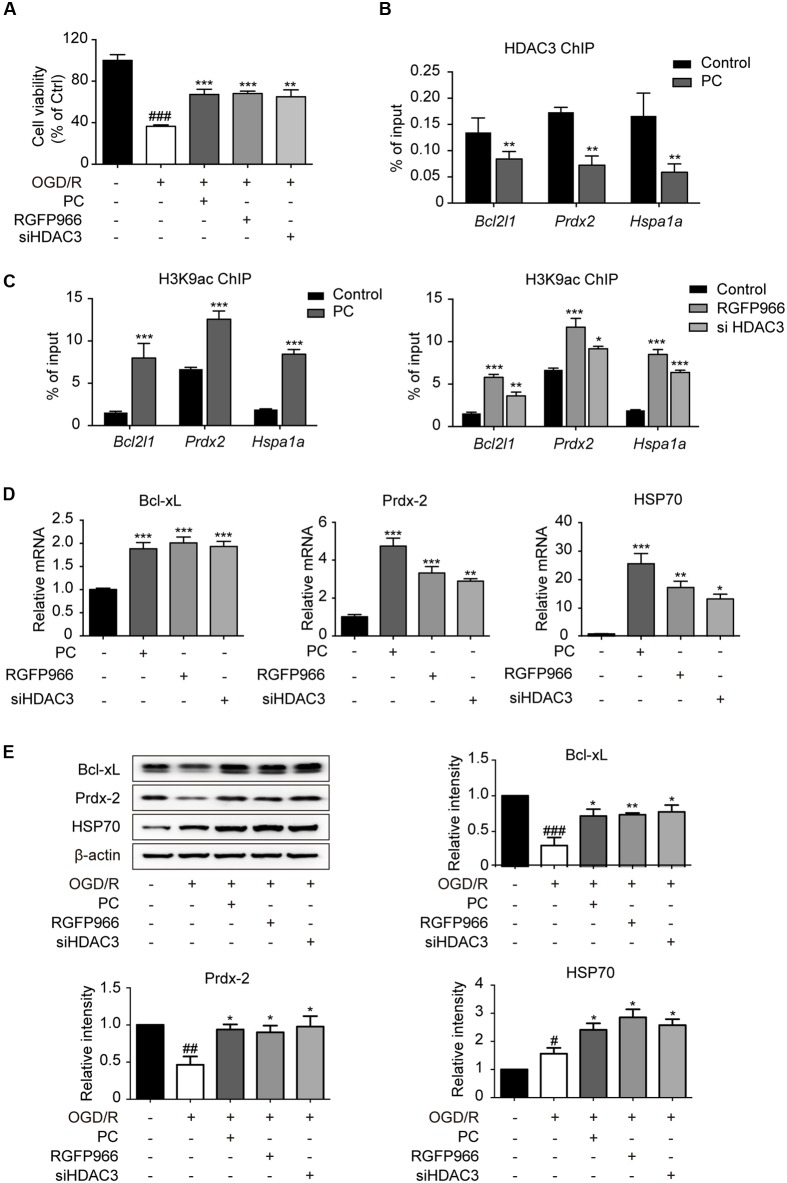
**Diminished recruitment of HDAC3 to the promoter region following PC potentiated the transcriptional initiation of oxidation relative genes. (A)** For non-PC groups, cortical neurons at 7 DIV were pretreated with RGFP966 (2 μM) for 2 h, or transfected with siRNA targeting HDAC3 at 5.5 DIV 36 h in advance. For PC group, PC was induced at 6 DIV followed by reoxygenation. All groups underwent OGD/R at the same time at 7 DIV. At the end of the OGD/R period, cell viability was assessed using an MTT assay and calculated as the percentage of the non-treatment control. ^###^*p* < 0.001 versus control; ^∗∗^*p* < 0.01, ^∗∗∗^*p* < 0.001 versus OGD/R, ANOVA. Primary cultured neurons were subjected to PC or pretreated with RGFP966 for 2 h at 7 DIV, or transfected with siRNA targeting HDAC3 at 5.5 DIV 36 h in advance. Samples for ChIP were collected right after PC or RGFP966 and siHDAC3 treatment **(B,C)**. For mRNA test samples were collected 6 h after PC followed by reoxygenation **(D)**. **(B)** ChIP with HDAC3 antibodies was conducted followed by qPCR analysis using primers for HDAC3 binding sites near the promoter of the indicated genes. **(C)** Acetylation of H3K9 near the HDAC3-targeting gene promoters were analyzed by ChIP-qPCR. **(D)** RT-qPCR analysis for indicated genes. ^∗^*p* < 0.05, ^∗∗^*p* < 0.01, ^∗∗∗^*p* < 0.001 versus control, Student’s *t*-test and ANOVA. **(E)** Neurons were induced with PC, pretreated with RGFP966 or siHDAC3 and then exposed to OGD/R (the same procedure with **A**). The expression of oxidative relative genes was analyzed at the protein levels. Cell lysates were analyzed with specific antibodies. Data are presented as means ± SEM from three independent experiments. ^#^*p* < 0.05, ^##^*p* < 0.01, ^###^*p* < 0.001 versus control; ^∗^*p* < 0.05, ^∗∗^*p* < 0.01 versus OGD/R, ANOVA.

Concomitant with the data *in vivo*, the reduction in cell viability elicited by OGD/R was blocked by both RGFP966 pretreatment and HDAC3 knock-down in primary cultured cortical neurons, as determined by the MTT assay (**Figure 3A**). To further elaborate the underlying role of HDAC3 in PC, data mining linking HDAC3 targeting genes (Feng et al., 2011) and ischemia was performed and determined a group of associated genes. ChIP analysis revealed that promoter occupancy of HDAC3 on *Hspa1a*, *Prdx2* and *Bcl2l1* were markedly reduced in response to PC, and concomitantly H3K9 acetylation levels were significantly elevated. Furthermore, RGFP966 pretreatment or HDAC3 knock-down also induced increases in H3K9 acetylation near the HDAC3 binding sites of indicated genes (**Figures 3B,C**). Consistently the expression of indicated genes increased significantly in PC, RGFP966 treatment or HDAC3 knock-down (**Figure 3D**). We next exposed primary neurons to injurious OGD/R with the same procedure in **Figure 3A**. Western blot analysis showed that OGD/R slightly induced HSP70 expression, but suppressed Bcl-xL and Prdx-2 expression, while RGFP966 treatment or HDAC3 knock-down, consistent with PC, enhanced expression of all indicated genes compared with OGD/R (**Figure 3E**, Supplementary Figure S6). Taken together, we have demonstrated that the diminished recruitment of HDAC3 observed in PC led to up-regulation of HDAC3 related genes associated with neuroprotection.

### Inhibition of Calpain1/Calpain2 Blocked the Reduction of HDAC3 Nuclear Localization Following PC

**FIGURE 4:**
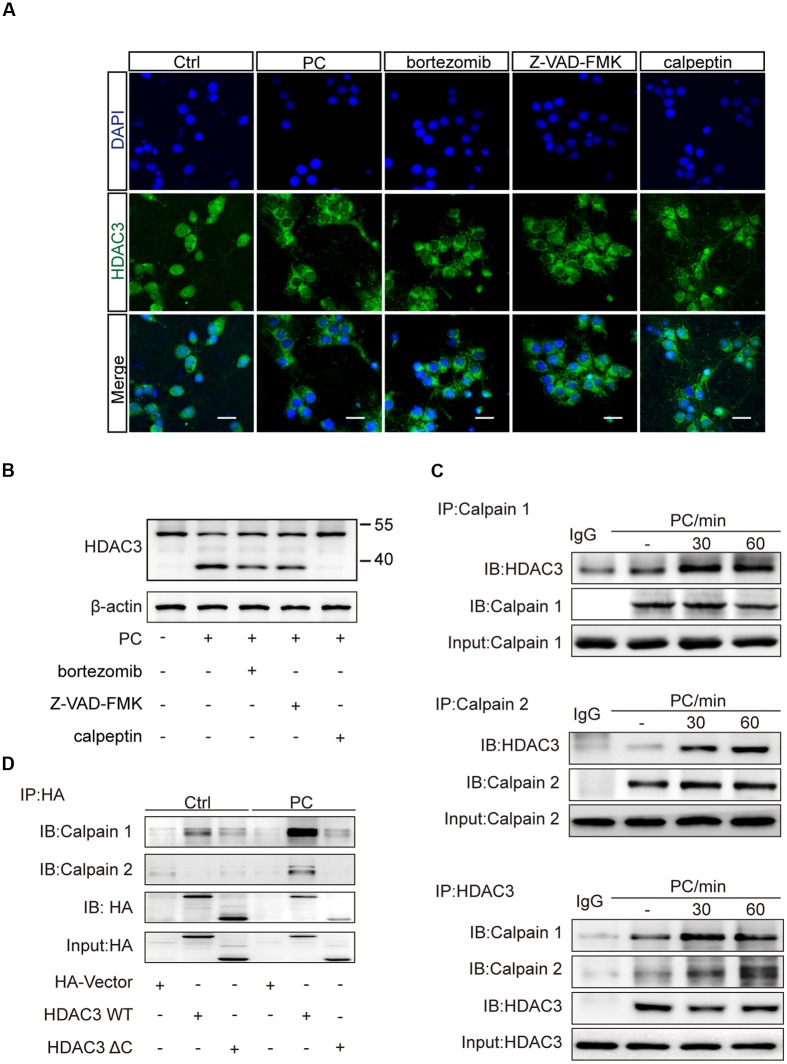
**Inhibition of calpains rescued the reduction of nuclear localization of HDAC3 following PC in neurons. (A)** Primary cultured neurons were treated with bortezomib (4 nM), Z-VAD-FMK (50 nM) or calpeptin (5 μM) for 2 h prior to PC. Immunohistochemistry was conducted after PC. HDAC3 localization was visualized using immunofluorescence. Scale bars: 20 μm. **(B)** Cleavage products were analyzed by immunoblotting with an antibody against the N-terminus of HDAC3 (ab47237, Abcam) after PC. **(C)** Bands of HDAC3 were obtained by immunoprecipitating calpain1 or calpain2 and immunoblotting with the anti-HDAC3 antibody. Bands of calpain1 and calpain2 were obtained by immunoprecipitating HDAC3 and immunoblotting with the anti-calpain1 and anti-calpain2 antibodies respectively. **(D)** Plasmids encoding HA-tag, HA-HDAC3 WT, HA-HDAC3 ΔC were transfected into PC12 cells and PC was performed 36 h later. Whole cell lysates were immunoprecipitated with anti-HA antibody after PC and immunoblotted with anti-calpain1 or anti-calpain2 antibodies.

Subcellular localization of HDAC3 was shown to be controlled by protein-protein interaction or proteolytic cleavage (Karagianni and Wong, 2007). The unique C-terminus of HDAC3 is essential for nuclear localization and assembly into co-repression complexes (Yang et al., 2002). Several cleavages of HDAC3 by different proteases, which are activated with temporal specificity in the process of cerebral ischemia, are involved in regulating the cellular distribution of HDAC3. We pretreated neurons with three major proteolytic pathway inhibitors including bortezomib (proteasome inhibitor), Z-VAD-FMK (pan-caspase inhibitor), and calpeptin (calpain inhibitor). PC was then conducted. Immunofluorescence results showed that calpeptin pretreatment, but not bortezomib or Z-VAD-FMK rescued the reduction of nuclear HDAC3 (**Figure 4A**). Western blot analysis with whole cell lysates showed a lower band of HDAC3 in PC treated neurons, suggesting a cleavage of HDAC3 induced by PC (**Figure 4B**). Furthermore, immunoprecipitation was performed with anti-HDAC3 or anti-calpain1/calpain2 antibodies. We found that direct interactions between HDAC3 and calpain1/calpain2 were strengthened after PC (**Figure 4C**). To further identify the interaction domain between HDAC3 and calpain, we constructed HA-tagged WT HDAC3 and truncated HDAC3 (aa1-312, HDAC3 ΔC) plasmids. Consistent with previous reports (Yang et al., 2002), HA-HDAC3 WT displayed both cytoplasmic and nuclear distributions, while HA-HDAC3 ΔC specifically localized in the cytoplasm of neurons (Supplementary Figure S3). A PC12 cell line was transfected with HDAC3 constructs and underwent PC. As expected, PC significantly increased the interaction of HA-HDAC3 WT with calpains, while truncated mutation, HA-HDAC3 ΔC, reduced the interaction with calpains after PC compared with control group (**Figure 4D**), indicating that the interaction domain may lie in the C-terminal region of HDAC3. From these results we concluded that blockade of HDAC3 nuclear localization following PC treatment resulted from the C-terminal cleavage of HDAC3 mediated by calpain1/calpain2.

### Calpeptin Blocked the Attenuated Nuclear Localization of HDAC3 and the Neuroprotective Effect of PC *In vivo*

**FIGURE 5:**
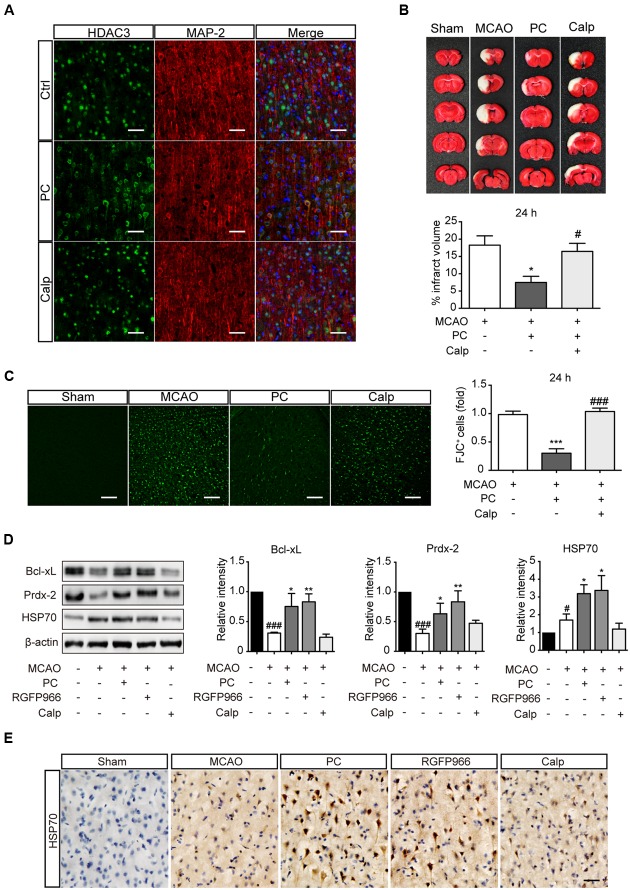
**Calpeptin pretreatment blocked the protective effect of PC *in vivo*. (A)** Representative images of HDAC3 subcellular localization in cortex. Scale bar: 50 μm. **(B)** Representative images of TTC staining from each group are shown. Infarct volume percentage from each slice were determined (*n* = 8–10). ^∗^*p* < 0.05 versus MCAO group, ^#^*p* < 0.05 versus PC group, ANOVA. **(C)** Representative images of Fluoro-Jade C staining and the numbers of degenerating neurons (over 30 slices from six rats per group). ^∗∗∗^*p* < 0.001 versus MCAO group, ^###^*p* < 0.001 versus PC group, ANOVA. **(D)** Expression levels of Prdx-2, Bcl-xL and HSP70 in each group (*n* = 3–4) 24 h after MCAO. ^#^*p* < 0.05, ^###^*p* < 0.001 versus Sham group; ^∗^*p* < 0.05, ^∗∗^*p* < 0.01 versus MCAO group, ANOVA. **(E)** Representative images of Cerebral HSP70 immunohistochemistry. Scale bar: 50 μm. Calp, calpeptin.

Rats were treated with calpeptin 6 h prior to surgery. To determine the subcellular localization of HDAC3, brain sections were obtained 2 h after PC. Immunofluorescence results showed that calpeptin pretreatment blocked the reduction of nuclear HDAC3 induced by PC (**Figure 5A**). For pathological assessment, rats subjected to MCAO were sacrificed 24 h or 7 d later (Supplementary Figure S2B). TTC staining (**Figure 5B**) and FJC immunoreactivity (**Figure 5C**) revealed that calpeptin had significantly attenuated the protective effect of PC 24 h after MCAO, consistent with results observed 7 days after MCAO (Supplementary Figure S4). Activated astrocytes and microglia were observed in MCAO and calpeptin group compared with PC group (Supplementary Figures S5D,E). Furthermore, we compared the expression level of indicated genes in all experimental groups, including the RGFP966 treatment group. Specimens were obtained 24 h after MCAO. Consistent with results *in vitro*, the levels of Prdx-2, Bcl-xL and HSP70 were significantly elevated in PC and RGFP966 groups compared with the MCAO group, while pretreatment with calpeptin attenuated the expression enhancement of PC (**Figure 5D**). HSP70 regulation was further assessed by immunohistochemistry, which revealed that HSP70 expression was enhanced by PC and RGFP966 treatment, whereas its expression was blocked by calpeptin pretreatment (**Figure 5E**).

## Discussion

Ischemic PC has been studied in order to develop new therapeutic approaches to benefit patients with stroke (Dirnagl et al., 2003; Gidday, 2006; Stetler et al., 2014). The present study provides evidence that HDAC3 inhibition may have a role in PC the brain against ischemia. We found that the reduction of HDAC3 nuclear localization following PC resulted in elevated acetylation of H3K9. Acting as one of the leading causes of PC, release of the HDAC3 co-repressor complex facilitated the transcription of relevant genes including *Hspa1a*, *Bcl2l1*, and *Prdx2*, which contributed to the endogenous protection elicited by PC. Further, we showed that C-terminal cleavage of HDAC3 by calpain underlies the failure of HDAC3 nuclear localization, and inhibition of calpain attenuated the PC protection exerted by HDAC3 inhibition.

There has been increasing interest in targeting epigenetic process as potential treatment for stroke (Langley et al., 2009; Aune et al., 2015). Several HDAC inhibitors such as VPA (Kim et al., 2007; Wang et al., 2012b), TSA (Yildirim et al., 2008; Wang et al., 2012a) and SAHA (Faraco et al., 2006) have previously shown protective effects against cerebral ischemia. Meanwhile, epigenetic modification is believed to play a fundamental role in ischemic tolerance. Yet the precise roles of HDACs in PC remains less clearly understood. Notably, it has been reported that total histone-H3 acetylation was down-regulated in cerebral ischemic injury but up-regulated following PC both *in vitro* and *in vivo* (Yildirim et al., 2014). Another study revealed a significant increase in retinal acetyl histone-H3 labeling after PC but a pronounced decrease after ischemia (Fan et al., 2016). In our study we further illustrated the time course of histone modification in both PC and OGD/R *in vitro*. Acetyl-H3K9 was elevated within 2 h following PC and returned to normal levels 6 h later (**Figure 1C**), while it was decreased profoundly 12 h after OGD followed by reoxygenation (Supplementary Figures S1A,B). Different mechanisms underlying the opposite regulation have been proposed. In the retina the deacetylase activity of HDAC1/2 displayed an increase in ischemia but a decrease following PC, which was closely correlated with histone acetylation modification (Fan et al., 2016). Whereas in the brain, a decrease in histone H3 acetylation was observed for 6 h after MCAO, strikingly without concomitant change in HAT or HDAC activity, which might be associated with decreased acetyl-CoA contents after ischemia (Faraco et al., 2006). In the brain following PC, increased histone acetylation was reported to result from enhanced CBP recruitment at the promoter of the neuroprotective gene *gelsolin* (Yildirim et al., 2014). Here, we reported that additional regulation is involved in this process, whereby cleaved HDAC3 is distributed in the cytoplasm and reducing the recruitment to targeted genes following PC, which consequently elevated the level of acetyl-H3K9. All these finding present a new perspective in explaining the protective role of HDAC inhibitors that reinstatement of the decreased histone acetylation level may exert the PC protection following ischemic injury.

Numerous studies have identified the mechanisms of PC against injurious ischemia. Gene profile analysis demonstrated that PC induced an extensive down-regulation of genes responsible for metabolic pathways and ion-channel activity, indicating lowered cellular activity (Stenzel-Poore et al., 2003, 2007). Similar regulation is implicated in the response to resveratrol treatment, which functions via the activation of SIRT1 (NAD-dependent deacetylase sirtuin-1; Raval et al., 2006; Koronowski et al., 2015). HIF-1-alpha also plays a fundamental role in adapting the metabolism to hypoxic conditions via transcriptional regulation (Prass et al., 2003; Tang et al., 2006). Apart from the known effectors that suppress energy turnover, cumulative evidence has suggested that epigenetic modulators may be involved in PC paradigms. The volume of ischemic tissue was reduced by the inhibition of DNA methylation and histone acetylation, which elicited a reprogramming of ischemic tolerance (Thompson et al., 2013). In our study, ChIP assays have revealed that the induction of PC, HDAC3 knock-down and HDAC3 inhibition elevated the histone acetylation level of related gene including *Hspa1a*, *Bcl2l1*, and *Prdx2*, and were further permissive to transcriptional initiation. Notably, over 12-fold up-regulation of HSP70 transcription was observed 12 h in PC, siHDAC3 and RGFP966 treated cortical neurons 6 h after PC induction or mimicking. It has been reported that HSP70 was induced in the penumbra, but not core, of the ischemic region, as well as in neurons which survive PC (Masada et al., 2001). Delaying administration of HSP70 by 2.25 and 3 h after the onset of ischemia significantly decreased infarct volume by 68% (Zhan et al., 2010). Bcl-xL is required for neurite outgrowth and potentially inhibits programmed cell death through changing the topology of the mitochondrial membrane (Park et al., 2015). Peroxiredoxin, an antioxidant enzyme, protects cells from the insults due to free radical accumulation (Finkel and Holbrook, 2000). These observations suggest that like other methods of manipulating PC, HDAC3 inhibition triggers signaling cascades in neurons leading to increased levels of free radical species and superoxide clearance, inhibition of the endogenous cell apoptosis program, and improved overall protein integrity, which shield the brain from ischemic injury.

In our study we demonstrated that calpain-mediated C-terminus cleavage of HDAC3 was responsible for the absence of HDAC3 in nuclei following PC. HDAC3 is a member of the class I HDAC family (HDAC1, HDAC2, HDAC3, and HDAC8), which share homology with Rpd3 from budding yeast (Yang and Seto, 2008). The unique C-terminus of HDAC3 is essential for its deacetylase activity and nuclear localization (Yang et al., 2002). Several ways of HDAC3 cleavage have been reported to affect its distribution. Etoposide treatment resulted in the cleavage of HDAC3 by caspase-3 and its subsequent cytoplasmic accumulation (Choi et al., 2015). Following treatment with FasL or ultraviolet irradiation, HDAC3 was cleaved in an indirect caspase-dependent manner and subsequently distributed in the cytoplasm (Escaffit et al., 2007). In the present study, minimum amount of interaction between HDAC3 and calpains were observed under normal conditions. While following PC and pretreatment with different protease inhibitors, we confirmed HDAC3 was cleaved by calpains, but not caspase or proteasome. From our results we cannot determine the difference in cleavage participation between calpain1 and calpain2. Through truncation research, we confirmed that the non-conserved C-terminal region of HDAC3 is required for the interaction of calpain with HDAC3, but the specific sequence remains to be illustrated. Calpains are a family of calcium-dependent proteases, which are profoundly and causally linked to post-ischemic insults (Bevers and Neumar, 2008), and calpain inhibitors have been investigated as potential therapeutics for cerebral ischemia (Koumura et al., 2008). The sustained calcium overload in stroke led to pathological activation of calpains, which resulted in extensive degradation of structural proteins and enzymes and neuronal cell death. The spectrin cleavages generated by calpains, SBDP150 and SBDP145, appeared 60 min after ischemia, and followed by a second expansion between 24 and 48 h after reperfusion (Neumar et al., 2001; Lin et al., 2013). These two steps of calpain activation may indicate that the controlled activation of calpain was induced at the beginning of ischemia, whereas sustained calcium overload resulted in pathological hyper-activation of calpains, which played predominant pathologic protease activity in vulnerable neurons following ischemia (Zhang et al., 2002; Liu et al., 2008). Regulated activation of calpain in the brain are involved in synaptic function and memory formation, but the mechanism underlying the controlled activation of calpain in PC has not been illustrated. We propose that firstly, PC is not an injurious stimulus, whereby transient signaling cascades may not lead to loss of Ca^2+^ homeostasis and pronounced activation of proteases. Secondly, other signaling pathways involved in PC may regulate and stabilize the homeostasis of Ca^2+^. For example, A1 adenosine receptors are reported to mediate the protection against infarction afforded by PC (Liu et al., 1991). And adenosine release from astrocytes down-regulated the synaptic activity level in transient hypoxia by negatively modulating the external or internal Ca^2+^ concentrations (Martín et al., 2007).

Here, we demonstrated that HDAC3 inhibition increased the resistance of the CNS to ischemic insult. Controlled activation of calpains in PC was involved in the cleavage of HDAC3, which reduced nuclear localization of HDAC3 and led to elevated acetylation of H3K9 and initiation of neuroprotective genes. Consistent with the role of HDAC inhibitors in neuroprotection, this study illustrates the role of specific HDAC subtypes in ischemic PC, and proposes new connections between PC and epigenetic effectors.

## Ethics Statement

The using of animals and experimental procedures were performed following the rules of the Association for Assessment and Accreditation of Laboratory Animal Care International and approved by the Institutional Animal Care and Use Committee of the Shanghai Institute of Material Medica, Chinese Academy of Sciences. No endangered animals were applied in this research.

## Author Contributions

XY and LF designed experiments. XY and QW performed most of the experiments. XY, QW, and LZ contributed to the animal behavior studies. XY, QW, and LZ analyzed the data. XY and LF wrote the paper.

## Conflict of Interest Statement

The authors declare that the research was conducted in the absence of any commercial or financial relationships that could be construed as a potential conflict of interest.

## Abbreviations

ChIP: chromatin immunoprecipetition
DIV: days *in vitro*
HAT: histone transacetylase
HDAC: histone deacetylase
MCAO: middle cerebral artery occlusion
MTT: 3-(4,5-dimethylthiazolyl)-2,5-diphenyltetrazolium bromide
OGD/R: oxygen-glucose deprivation and reoxygenation and restored energy supply
PC: preconditioning
TTC: 2,3,5-Triphenyltetrazolium chloride.

## References

[B1] Aune S. E. Herr D. J. Kutz C. J. Menick D. R. (2015). Histone deacetylases exert class-specific roles in conditioning the brain and heart against acute ischemic injury. *Front. Neurol.* 6:145 10.3389/fneur.2015.00145 PMC4485035 26175715

[B2] Bardai F. H. D’Mello S. R. (2011). Selective toxicity by HDAC3 in neurons: regulation by Akt and GSK3beta. *J. Neurosci.* 31 1746–1751. 10.1523/JNEUROSCI.5704-10.2011 21289184 PMC3711464

[B3] Bardai F. H. Verma P. Smith C. Rawat V. Wang L. D’Mello S. R. (2013). Disassociation of histone deacetylase-3 from normal huntingtin underlies mutant huntingtin neurotoxicity. *J. Neurosci.* 33 11833–11838. 10.1523/JNEUROSCI.5831-12.2013 23864673 PMC3713725

[B4] Bates E. A. Victor M. Jones A. K. Shi Y. Hart A. C. (2006). Differential contributions of *Caenorhabditis elegans* histone deacetylases to huntingtin polyglutamine toxicity. *J. Neurosci.* 26 2830–2838. 10.1523/JNEUROSCI.3344-05.2006 16525063 PMC6675170

[B5] Bevers M. B. Neumar R. W. (2008). Mechanistic role of calpains in postischemic neurodegeneration. *J. Cereb. Blood Flow Metab.* 28 655–673. 10.1038/sj.jcbfm.9600595 18073773

[B6] Butler R. Bates G. P. (2006). Histone deacetylase inhibitors as therapeutics for polyglutamine disorders. *Nat. Rev. Neurosci.* 7 784–796. 10.1038/nrn1989 16988654

[B7] Choi H. K. Choi Y. Park E. S. Park S. Y. Lee S. H. Seo J. (2015). Programmed cell death 5 mediates HDAC3 decay to promote genotoxic stress response. *Nat. Commun.* 6:7390 10.1038/ncomms8390 PMC4490383 26077467

[B8] Chuang D. M. Leng Y. Marinova Z. Kim H. J. Chiu C. T. (2009). Multiple roles of HDAC inhibition in neurodegenerative conditions. *Trends Neurosci.* 32 591–601. 10.1016/j.tins.2009.06.002 19775759 PMC2771446

[B9] Dirnagl U. Becker K. Meisel A. (2009). Preconditioning and tolerance against cerebral ischaemia: from experimental strategies to clinical use. *Lancet Neurol.* 8 398–412. 10.1016/S1474-4422(09)70054-7 19296922 PMC2668955

[B10] Dirnagl U. Iadecola C. Moskowitz M. A. (1999). Pathobiology of ischaemic stroke: an integrated view. *Trends Neurosci.* 22 391–397. 10.1016/S0166-2236(99)01401-0 10441299

[B11] Dirnagl U. Simon R. P. Hallenbeck J. M. (2003). Ischemic tolerance and endogenous neuroprotection. *Trends Neurosci.* 26 248–254. 10.1016/S0166-2236(03)00071-7 12744841

[B12] Escaffit F. Vaute O. Chevillard-Briet M. Segui B. Takami Y. Nakayama T. (2007). Cleavage and cytoplasmic relocalization of histone deacetylase 3 are important for apoptosis progression. *Mol. Cell. Biol.* 27 554–567. 10.1128/MCB.00869-06 17101790 PMC1800792

[B13] Fan J. Alsarraf O. Chou C. J. Yates P. W. Goodwin N. C. Rice D. S. (2016). Ischemic preconditioning, retinal neuroprotection and histone deacetylase activities. *Exp. Eye Res.* 146 269–275. 10.1016/j.exer.2016.03.026 27060376 PMC4893999

[B14] Faraco G. Pancani T. Formentini L. Mascagni P. Fossati G. Leoni F. (2006). Pharmacological inhibition of histone deacetylases by suberoylanilide hydroxamic acid specifically alters gene expression and reduces ischemic injury in the mouse brain. *Mol. Pharmacol.* 70 1876–1884. 10.1124/mol.106.027912 16946032

[B15] Feigin V. L. Forouzanfar M. H. Krishnamurthi R. Mensah G. A. Connor M. Bennett D. A. (2014). Global and regional burden of stroke during 1990–2010: findings from the Global Burden of Disease Study 2010. *Lancet* 383 245–255. 10.1016/S0140-6736(13)61953-4 24449944 PMC4181600

[B16] Feng D. Liu T. Sun Z. Bugge A. Mullican S. E. Alenghat T. (2011). A circadian rhythm orchestrated by histone deacetylase 3 controls hepatic lipid metabolism. *Science* 331 1315–1319. 10.1126/science.1198125 21393543 PMC3389392

[B17] Finkel T. Holbrook N. J. (2000). Oxidants, oxidative stress and the biology of ageing. *Nature* 408 239–247. 10.1038/35041687 11089981

[B18] Fisher M. Saver J. L. (2015). Future directions of acute ischaemic stroke therapy. *Lancet Neurol.* 14 758–767. 10.1016/s1474-4422(15)00054-x 26067128

[B19] Gidday J. M. (2006). Cerebral preconditioning and ischaemic tolerance. *Nat. Rev. Neurosci.* 7 437–448. 10.1038/nrn1927 16715053

[B20] Hunter A. Hatcher J. Virley D. Nelson P. Irving E. Hadingham S. (2000). Functional assessments in mice and rats after focal stroke. *Neuropharmacology* 39 806–816. 10.1016/S0028-3908(99)00262-2 10699446

[B21] Johnston S. C. Mendis S. Mathers C. D. (2009). Global variation in stroke burden and mortality: estimates from monitoring, surveillance, and modelling. *Lancet Neurol.* 8 345–354. 10.1016/S1474-4422(09)70023-7 19233730

[B22] Karagianni P. Wong J. (2007). HDAC3: taking the SMRT-N-CoRrect road to repression. *Oncogene* 26 5439–5449. 10.1038/sj.onc.1210612 17694085

[B23] Kim H. J. Rowe M. Ren M. Hong J. S. Chen P. S. Chuang D. M. (2007). Histone deacetylase inhibitors exhibit anti-inflammatory and neuroprotective effects in a rat permanent ischemic model of stroke: multiple mechanisms of action. *J. Pharmacol. Exp. Ther.* 321 892–901. 10.1124/jpet.107.120188 17371805

[B24] Koronowski K. B. Dave K. R. Saul I. Camarena V. Thompson J. W. Neumann J. T. (2015). Resveratrol preconditioning induces a novel extended window of ischemic tolerance in the mouse brain. *Stroke* 46 2293–2298. 10.1161/STROKEAHA.115.009876 26159789 PMC4519394

[B25] Koumura A. Nonaka Y. Hyakkoku K. Oka T. Shimazawa M. Hozumi I. (2008). A novel calpain inhibitor,((1S)-1 ((((1S)-1-benzyl-3-cyclopropylamino-2, 3-di-oxopropyl) amino) carbonyl)-3-methylbutyl) carbamic acid 5-methoxy-3-oxapentyl ester, protects neuronal cells from cerebral ischemia-induced damage in mice. *Neuroscience* 157 309–318. 10.1016/j.neuroscience.2008.09.007 18835333

[B26] Langley B. Brochier C. Rivieccio M. A. (2009). Targeting histone deacetylases as a multifaceted approach to treat the diverse outcomes of stroke. *Stroke* 40 2899–2905. 10.1161/STROKEAHA.108.540229 19478231

[B27] Lin Y. Zhang J.-C. Fu J. Chen F. Wang J. Wu Z.-L. (2013). Hyperforin attenuates brain damage induced by transient middle cerebral artery occlusion (MCAO) in rats via inhibition of TRPC6 channels degradation. *J. Cereb. Blood Flow Metab.* 33 253–262. 10.1038/jcbfm.2012.164 23149561 PMC3564196

[B28] Liu G. Thornton J. Van Winkle D. Stanley A. Olsson R. Downey J. (1991). Protection against infarction afforded by preconditioning is mediated by A1 adenosine receptors in rabbit heart. *Circulation* 84 350–356. 10.1161/01.CIR.84.1.350 2060105

[B29] Liu J. Liu M. C. Wang K. (2008). Calpain in the CNS: from synaptic function to neurotoxicity. *Sci. Signal.* 1:re1 10.1126/stke.114re1 18398107

[B30] Longa E. Z. Weinstein P. R. Carlson S. Cummins R. (1989). Reversible middle cerebral artery occlusion without craniectomy in rats. *Stroke* 20 84–91. 10.1161/01.STR.20.1.84 2643202

[B31] Malvaez M. McQuown S. C. Rogge G. A. Astarabadi M. Jacques V. Carreiro S. (2013). HDAC3-selective inhibitor enhances extinction of cocaine-seeking behavior in a persistent manner. *Proc. Natl. Acad. Sci. U.S.A.* 110 2647–2652. 10.1073/pnas.1213364110 23297220 PMC3574934

[B32] Martín E. D. Fernández M. Perea G. Pascual O. Haydon P. G. Araque A. (2007). Adenosine released by astrocytes contributes to hypoxia-induced modulation of synaptic transmission. *Glia* 55 36–45. 10.1002/glia.20431 17004232

[B33] Masada T. Hua Y. Xi G. Ennis S. R. Keep R. F. (2001). Attenuation of ischemic brain edema and cerebrovascular injury after ischemic preconditioning in the rat. *J. Cereb. Blood Flow Metab.* 21 22–33. 10.1097/00004647-200101000-00004 11149665

[B34] Moretti A. Ferrari F. Villa R. F. (2015). Pharmacological therapy of acute ischaemic stroke: achievements and problems. *Pharmacol. Ther.* 153 79–89. 10.1016/j.pharmthera.2015.06.004 26079382

[B35] Moskowitz M. A. Lo E. H. Iadecola C. (2010). The science of stroke: mechanisms in search of treatments. *Neuron* 67 181–198. 10.1016/j.neuron.2010.07.002 20670828 PMC2957363

[B36] Neumar R. W. Meng F. H. Mills A. M. Xu Y. A. Zhang C. Welsh F. A. (2001). Calpain activity in the rat brain after transient forebrain ischemia. *Exp. Neurol.* 170 27–35. 10.1006/exnr.2001.7708 11421581

[B37] Park H. A. Licznerski P. Alavian K. N. Shanabrough M. Jonas E. A. (2015). Bcl-xL is necessary for neurite outgrowth in hippocampal neurons. *Antioxid. Redox. Signal.* 22 93–108. 10.1089/ars.2013.5570 24787232 PMC4281845

[B38] Prass K. Scharff A. Ruscher K. Lowl D. Muselmann C. Victorov I. (2003). Hypoxia-induced stroke tolerance in the mouse is mediated by erythropoietin. *Stroke* 34 1981–1986. 10.1161/01.STR.0000080381.76409.B2 12829864

[B39] Raval A. P. Dave K. R. Perez-Pinzon M. A. (2006). Resveratrol mimics ischemic preconditioning in the brain. *J. Cereb. Blood Flow Metab.* 26 1141–1147. 10.1038/sj.jcbfm.9600262 16395277

[B40] Ren M. Leng Y. Jeong M. Leeds P. R. Chuang D. M. (2004). Valproic acid reduces brain damage induced by transient focal cerebral ischemia in rats: potential roles of histone deacetylase inhibition and heat shock protein induction. *J. Neurochem.* 89 1358–1367. 10.1111/j.1471-4159.2004.02406.x 15189338

[B41] Samantaray S. Knaryan V. H. Shields D. C. Cox A. A. Haque A. Banik N. L. (2015). Inhibition of calpain activation protects MPTP-Induced nigral and spinal cord neurodegeneration, reduces inflammation, and improves gait dynamics in mice. *Mol. Neurobiol.* 52 1054–1066. 10.1007/s12035-015-9255-6 26108182 PMC4558265

[B42] Schmitt H. M. Pelzel H. R. Schlamp C. L. Nickells R. W. (2014). Histone deacetylase 3 (HDAC3) plays an important role in retinal ganglion cell death after acute optic nerve injury. *Mol. Neurodegen* 9:1 10.1186/1750-1326-9-39 PMC4190472 25261965

[B43] Stenzel-Poore M. P. Stevens S. L. King J. S. Simon R. P. (2007). Preconditioning reprograms the response to ischemic injury and primes the emergence of unique endogenous neuroprotective phenotypes: a speculative synthesis. *Stroke* 38 2(Suppl.), 680–685. 10.1161/01.STR.0000251444.56487.4c 17261715

[B44] Stenzel-Poore M. P. Stevens S. L. Xiong Z. Lessov N. S. Harrington C. A. Mori M. (2003). Effect of ischaemic preconditioning on genomic response to cerebral ischaemia: similarity to neuroprotective strategies in hibernation and hypoxia-tolerant states. *Lancet* 362 1028–1037. 10.1016/S0140-6736(03)14412-1 14522533

[B45] Stetler R. A. Leak R. K. Gan Y. Li P. Zhang F. Hu X. (2014). Preconditioning provides neuroprotection in models of CNS disease: paradigms and clinical significance. *Prog. Neurobiol.* 114 58–83. 10.1016/j.pneurobio.2013.11.005 24389580 PMC3937258

[B46] Stevens S. L. Vartanian K. B. Stenzel-Poore M. P. (2014). Reprogramming the response to stroke by preconditioning. *Stroke* 45 2527–2531. 10.1161/STROKEAHA.114.002879 24938838 PMC4240276

[B47] Tang Y. Pacary E. Freret T. Divoux D. Petit E. Schumann-Bard P. (2006). Effect of hypoxic preconditioning on brain genomic response before and following ischemia in the adult mouse: identification of potential neuroprotective candidates for stroke. *Neurobiol. Dis.* 21 18–28. 10.1016/j.nbd.2005.06.002 16040250

[B48] Thompson J. W. Dave K. R. Young J. I. Perez-Pinzon M. A. (2013). Ischemic preconditioning alters the epigenetic profile of the brain from ischemic intolerance to ischemic tolerance. *Neurotherapeutics* 10 789–797. 10.1007/s13311-013-0202-9 23868468 PMC3805868

[B49] Wang B. Zhu X. Kim Y. Li J. Huang S. Saleem S. (2012a). Histone deacetylase inhibition activates transcription factor Nrf2 and protects against cerebral ischemic damage. *Free Radic. Biol. Med.* 52 928–936. 10.1016/j.freeradbiomed.2011.12.006 22226832 PMC6010182

[B50] Wang Z. Tsai L. K. Munasinghe J. Leng Y. Fessler E. B. Chibane F. (2012b). Chronic valproate treatment enhances postischemic angiogenesis and promotes functional recovery in a rat model of ischemic stroke. *Stroke* 43 2430–2436. 10.1161/STROKEAHA.112.652545 22811460 PMC3429729

[B51] Xie K. Q. Zhang L. M. Cao Y. Zhu J. Feng L. Y. (2009). Adenosine A(1) receptor-mediated transactivation of the EGF receptor produces a neuroprotective effect on cortical neurons in vitro. *Acta Pharmacol. Sin.* 30 889–898. 10.1038/aps.2009.80 19574994 PMC4006641

[B52] Yang W. M. Tsai S. C. Wen Y. D. Fejer G. Seto E. (2002). Functional domains of histone deacetylase-3. *J. Biol. Chem.* 277 9447–9454. 10.1074/jbc.M105993200 11779848

[B53] Yang X. J. Seto E. (2008). The Rpd3/Hda1 family of lysine deacetylases: from bacteria and yeast to mice and men. *Nat. Rev. Mol. Cell Biol.* 9 206–218. 10.1038/nrm2346 18292778 PMC2667380

[B54] Yildirim F. Gertz K. Kronenberg G. Harms C. Fink K. B. Meisel A. (2008). Inhibition of histone deacetylation protects wildtype but not gelsolin-deficient mice from ischemic brain injury. *Exp. Neurol.* 210 531–542. 10.1016/j.expneurol.2007.11.031 18234195

[B55] Yildirim F. Ji S. Kronenberg G. Barco A. Olivares R. Benito E. (2014). Histone acetylation and CREB binding protein are required for neuronal resistance against ischemic injury. *PLoS ONE* 9:e95465 10.1371/journal.pone.0095465 PMC3991684 24748101

[B56] Zhan X. Ander B. P. Liao I. H. Hansen J. E. Kim C. Clements D. (2010). Recombinant Fv-Hsp70 protein mediates neuroprotection after focal cerebral ischemia in rats. *Stroke* 41 538–543. 10.1161/STROKEAHA.109.572537 20075343 PMC2957177

[B57] Zhang C. Siman R. Xu Y. A. Mills A. M. Frederick J. R. Neumar R. W. (2002). Comparison of calpain and caspase activities in the adult rat brain after transient forebrain ischemia. *Neurobiol. Dis.* 10 289–305. 10.1006/nbdi.2002.0526 12270691

