# Supplementary material for: Inhibition of Histone Deacetylase 3 (HDAC3) Mediates Ischemic Preconditioning and Protects Cortical Neurons against Ischemia in Rats
Source: Front Mol Neurosci. 2016 Nov 28;9:131. doi: 10.3389/fnmol.2016.00131 (PMC5124709; doi:10.3389/fnmol.2016.00131)
Supplement: Supplementary file 1 [file Data_Sheet_1.DOCX]

**SUPPLEMENTAL INFORMATION**

**Inhibition of Histone Deacetylase 3 (HDAC3) Mediates Ischemic Preconditioning and Protects Cortical Neurons against Ischemia in Rats**

Xiaoyu Yang, Qimei Wu, Lei Zhang, and Linyin Feng^*^

CAS Key Laboratory of Receptor Research, Chinese Academy of Sciences, Shanghai Institute of Materia Medica, 555 Zu Chong Zhi Road, Shanghai, 201203, P. R. China

* Correspondence: Professor Linyin Feng

Shanghai Institute of Materia Medica

Chinese Academy of Sciences

Phone: 86-21-50806810

Fax: 86-21-50806810

Email: [lyfeng@simm.ac.cn](mailto:lyfeng@simm.ac.cn)

**Supplementary Experimental procedures**

Fluoro-Jade C staining and immunofluorescence

For double-labeling experiments, sections were stained first with immunofluorescence. Briefly, sections were blocked in phosphate buffered saline with 10% normal goat serum and incubated in rabbit anti-GFAP(Merck-Millipore), rabbit anti-Iba (Dako) overnight at 4 °C. Subsequently, the sections were rinsed in PBS, and incubated with Alexa 555-conjugated goat anti-rabbit IgG (Thermo Fisher Scientific) for 1 h at 37 °C. After washing, sections were mounted on positively charged slides, dried at 50 °C and subjected Fluoro-Jade C staining. Pretreatment with ethanol tended to reduce the immunoreactivity and therefore was omitted, the slides were incubated in 0.06% potassium permanganate solution for 7 min and FJC staining for 20 min. The slides were washed and then coverslipped with DPX (Sigma). Images were obtained using Olympus FV1000 confocal microscope.

**
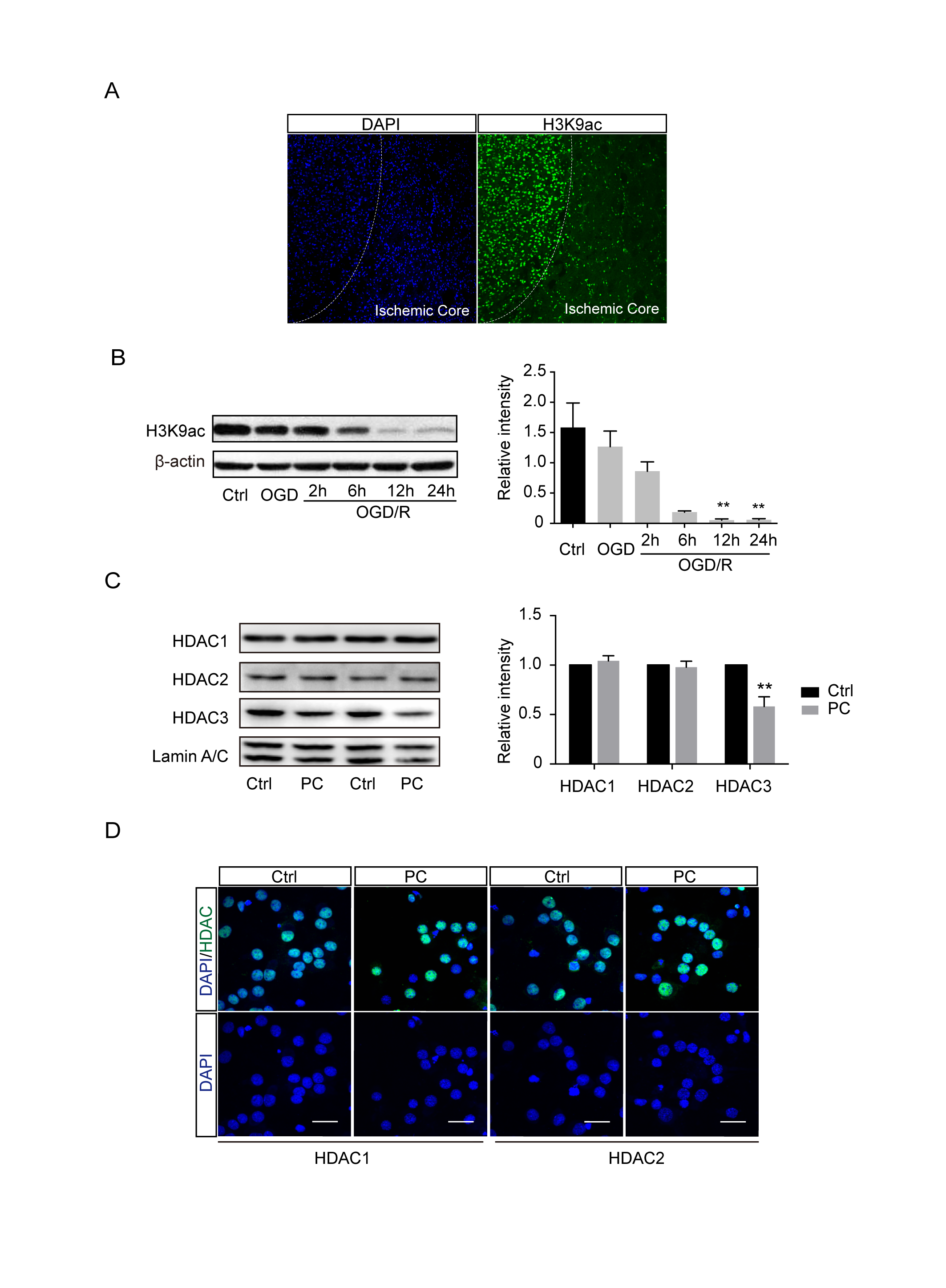
**

**Supplementary Figure S1. Histone modification and regulation of HDACs in ischemia and preconditioning models (A)** Immunofluorescence of H3K9ac were conducted with brain sections obtained 24 h after MCAO. The ischemic core was outlined in the right side of the ischemic striatum. **(B)** Primary cultured rat corical neurons were conducted to OGD (90 min) treatment. The time course of H3K9 acetylation after reoxygenation were obtained from whole cell lysates and quantified. Data are mean ± SEM from three independent experiments. (** P < 0.01 versus control group) **(C)** Primary cultured rat corical neurons were conducted to PC for 45 min followed by reoxygenation. Class I HDAC subtypes were assessed by western blot with nuclear lysates. Data are mean ± SEM. (n=4). Student’s t-test, **p<0.01. (**D)** Representative images of HDAC1 and HDAC2 subcellular localization visualized by immunofluorescence. Scale bar: 25 μm.

**
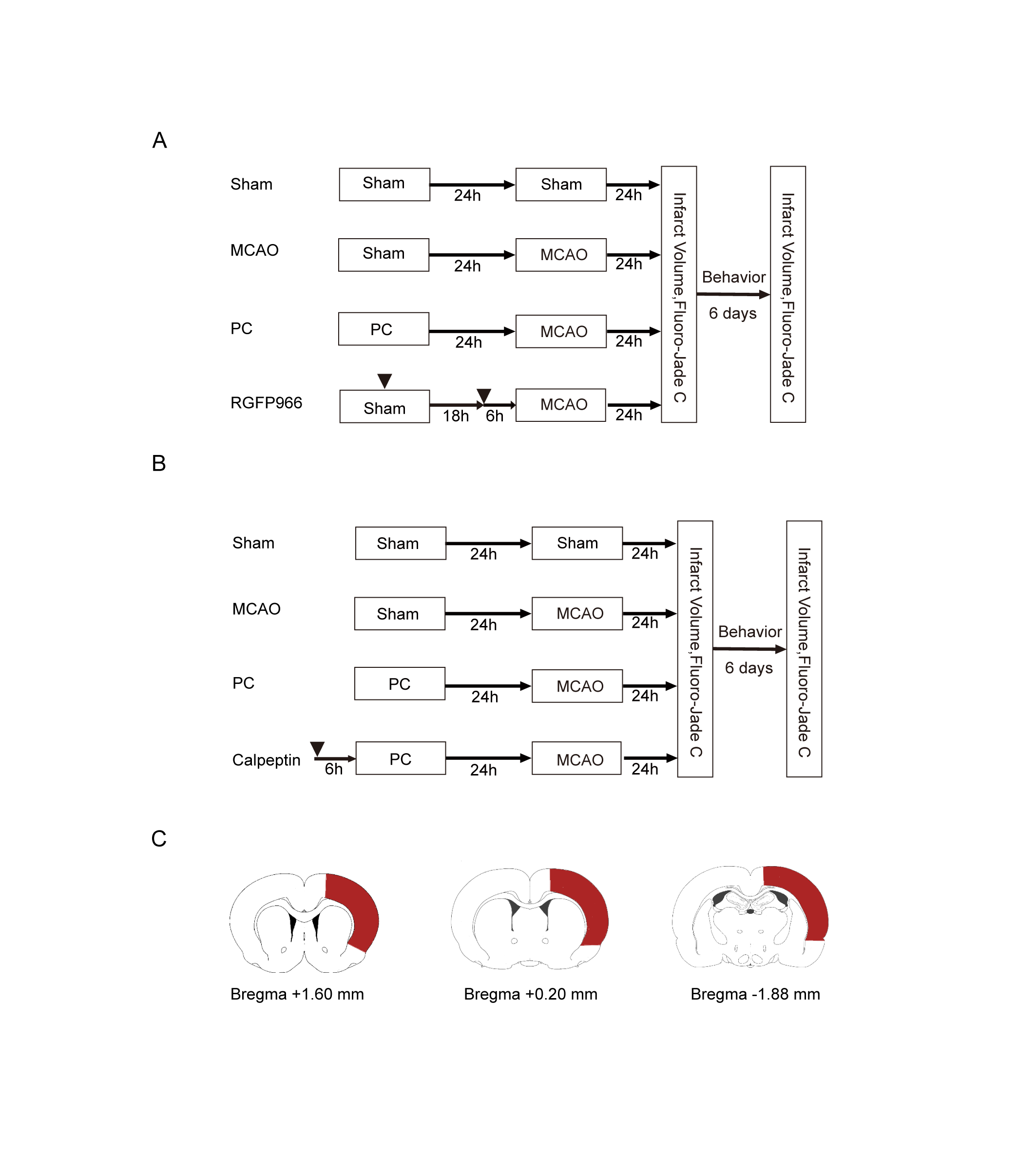
**

**Supplementary Figure S2. Schematic paragraph of MCAO experiment. (A, B)** Treatments are performed as described in Method. **(C)** Images for FJC statistics were randomly chosen from the shadowed areas in the schematic brain sections.

.
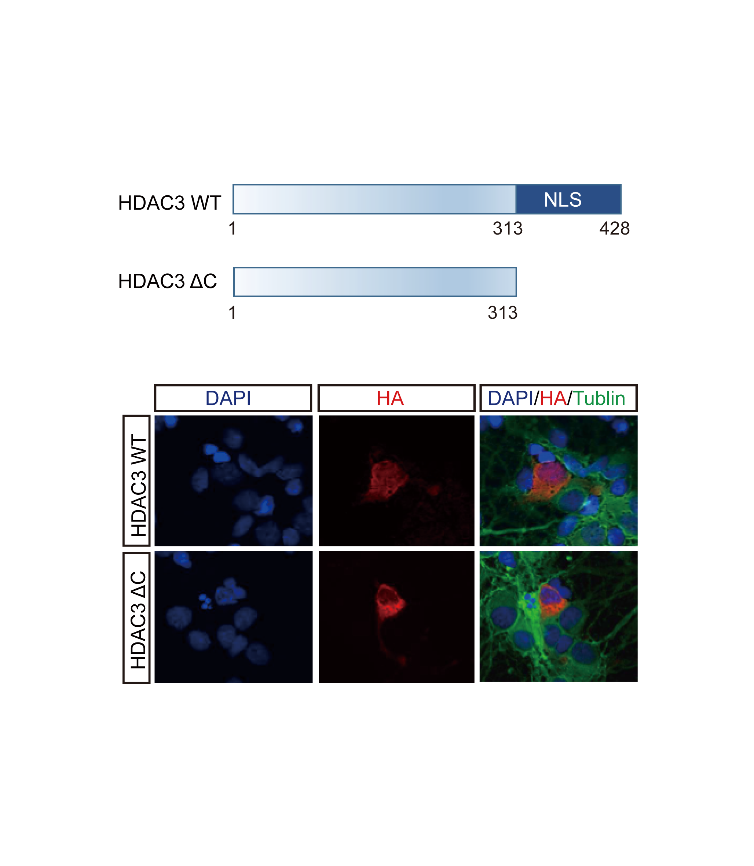


**Supplementary Figure S3. C-terminus truncated HDAC3 located in cytoplasm.** Primary cultured cortex neurons were transfected with HA-tagged HDAC3 and C-terminus truncated HDAC3.

**
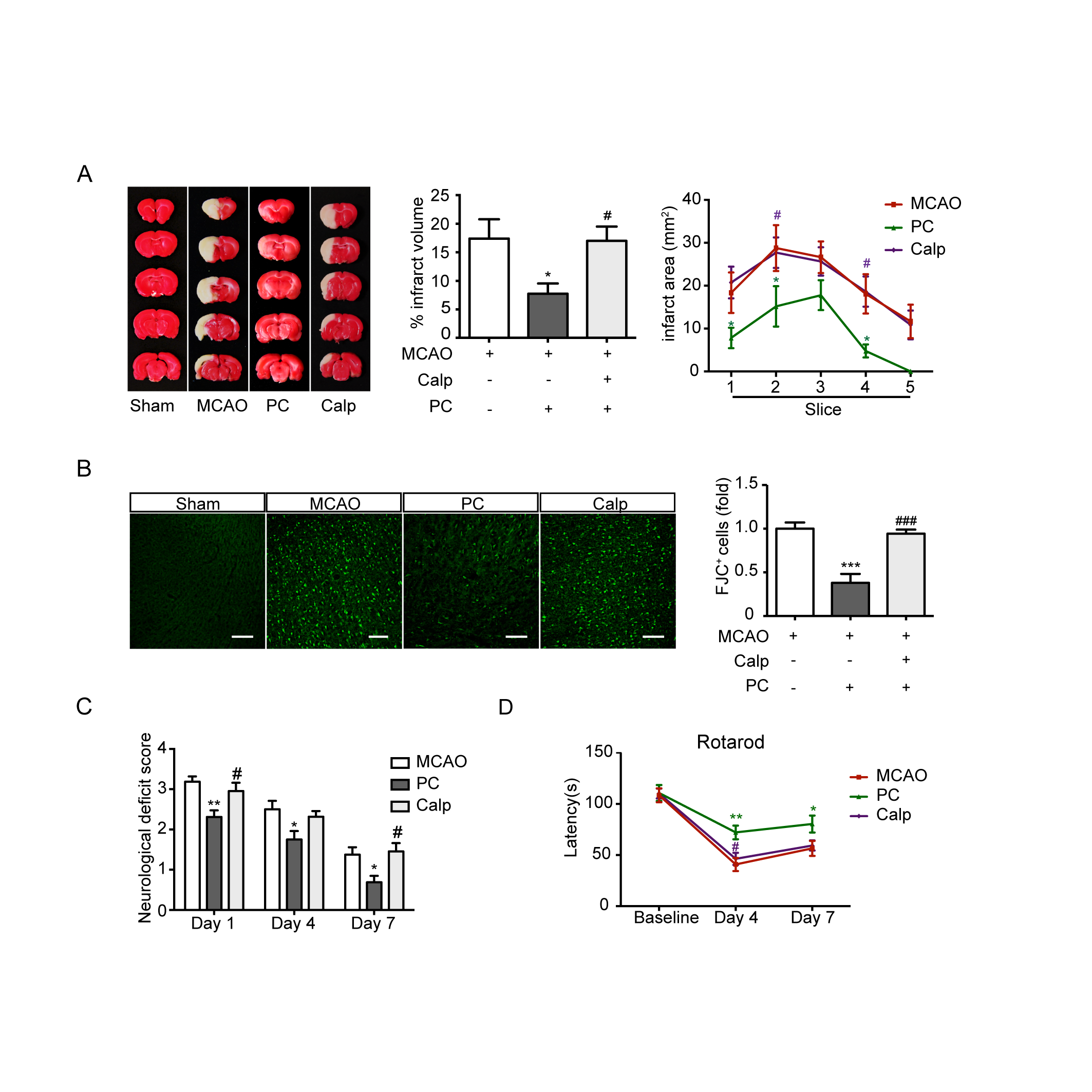
Supplementary Figure S4.** **Calpeptin pretreatment blockaded the protective effect of PC in 7 d assessment *in vivo*. (A)** Representative images of TTC staining from each group are shown. Infarct volume percentage and infarct area from each slice were determined (n=8-10). *p<0.05, ***p<0.001 versus MCAO group, #p<0.05 versus PC group, ANOVA. (**B)** Representative images of Fluoro-Jade C staining and the numbers of degenerating neurons (over 30 slices from 6 rats per group). ***p<0.001 versus MCAO group, ###p<0.001 versus PC group, ANOVA. (**C)** Functional outcomes were assessed with neurological deficit score (day 1, 4 and 7) and rotarod test (day 4 and 7) after MCAO (n=8-10). *p<0.05, **p<0.01 versus MCAO group, ANOVA.

**
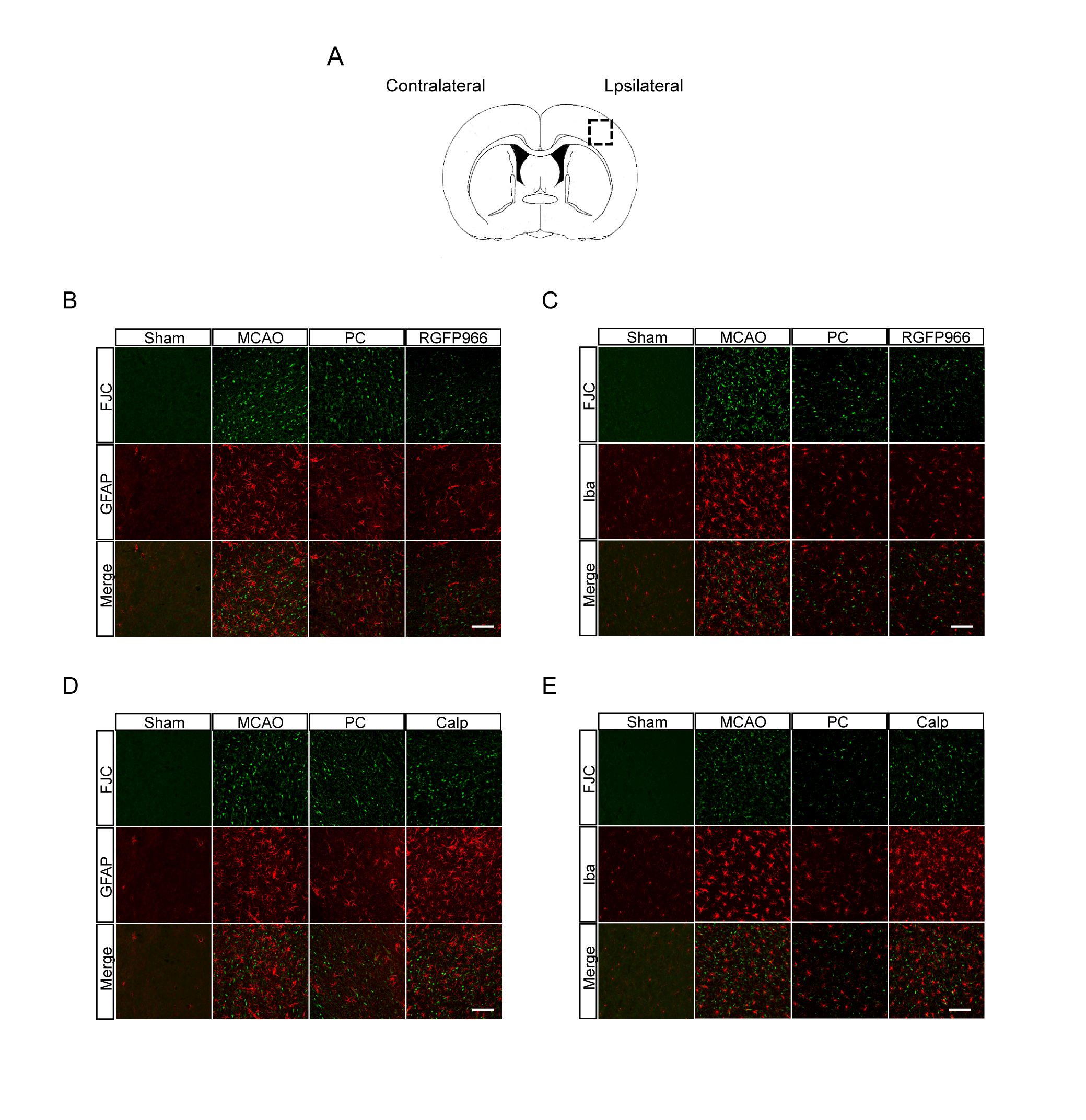
Supplementary Figure S5. Double labeling of FJC and astrocyte or microglia.** Sketch picture **(A)** showed the position of images obtained. Representative images of

counterstain of FJC with GFAP **(B, D)** or Iba-1 **(C, E)** with brain sections obtained 24 h after MCAO. Scale bar, 100 μm.

**Supplementary Figure S6. Effect of OGD/R on the transcription of HDAC3 targeted genes.** Primary cultured cortical neurons were subjected to OGD followed by 24 h reoxygenation and restore of energy. **(A)** ChIP with HDAC3 antibodies was conducted followed by qPCR analysis using primers for HDAC3 binding sites near the promoter of the indicated genes. **(B)** Acetylation of H3K9 near the HDAC3-targeting gene promoters were analyzed by ChIP-qPCR. **(C)** RT-qPCR analysis for indicated genes. *p<0.05, **p<0.01, ***p<0.001 versus control, Student’s t-test.

**Supplementary Table S1. Primers used in RT-qPCR.**

| RT-qPCR | Forward | Reverse |
| --- | --- | --- |
| HSP70 | GGCGCTCCAGGTGTGATCTA | GACTTGATTGCAGACCGAACGA |
| Prdx-2 | TTTAGCGACCACGCTGAGGAC | ACACGCCGTAATTCTGGGACA |
| Bcl-xL | AGCGAGCAGAAGCTGACACC | AAGGGCATTCTCACCTCTATCTCAC |
| Beta-actin | GGAGATTACTGCCCTGGCTCCTA | GACTCATCGTACTCCTGCTTGCTG |

**Supplementary Table S2. Primers used in ChIP-qPCR.**

| ChIP | Forward | Reverse |
| --- | --- | --- |
| Hsp1a1 | TGGTCTGATTCCCAAATGTCTCT | GCAAGGTAGCGGTCTCTGTATGT |
| Prdx2 | CTACCATCTCCACCGCACCT | CGGGCTTCCTTGGTCATCT |
| Bcl2l1 | TGGATGAAGGAACTAGATTGATGG | CCGAGACGCAAAAGGAGTG |
